# Emerging prediction methods for early diagnosis of necrotizing enterocolitis

**DOI:** 10.3389/fmed.2022.985219

**Published:** 2022-09-16

**Authors:** Siyuan Wu, Sijia Di, Tianjing Liu, Yongyan Shi

**Affiliations:** Department of Pediatrics, Shengjing Hospital of China Medical University, Shenyang, China

**Keywords:** necrotizing enterocolitis, early prediction, ultrasound, near-infrared spectroscopy, biomarkers, microbiota

## Abstract

Necrotizing enterocolitis (NEC) is a life-threatening disease of the digestive system that occurs in the neonatal period. NEC is difficult to diagnose early and the prognosis is poor. Previous studies have reported that abnormalities can be detected before the presentation of clinical symptoms. Based on an analysis of literature related to the early prediction of NEC, we provide a detailed review on the early prediction and diagnosis methods of NEC, including ultrasound, near-infrared spectroscopy, biomarkers, and intestinal microbiota. This review aimed to provide a reference for further research and clinical practice.

## Introduction

Necrotizing enterocolitis (NEC) is a life-threatening digestive system disease that occurs in the neonatal period. The incidence of NEC in neonates ranged from 2% to 7%, and the mortality rate was up to 21.9–38% (1). It is one of the leading causes of death in neonates, especially preterm infants. NEC is also associated with long-term complications, such as intestinal adhesion stenosis, short bowel syndrome, and developmental sensory and motor deficits (2, 3).

**Figure 1:**
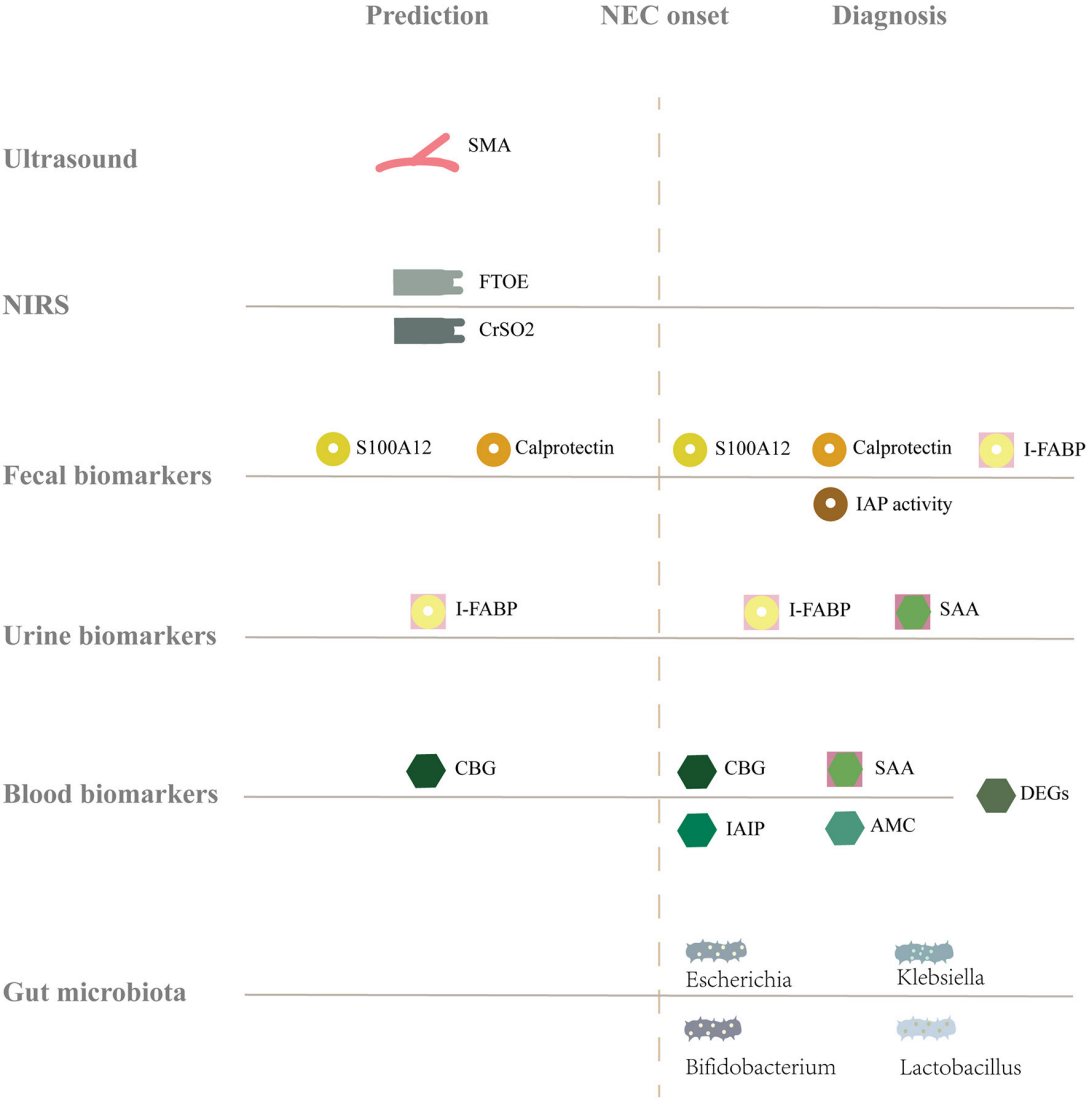
Summary of the full text. The gray solid line represents the baseline level of healthy infants, and above, below, and after the solid line represents the increase, decrease and change of markers, respectively. NEC, Necrotizing enterocolitis; SMA, Superior mesenteric artery; NIRS, Near-infrared spectroscopy; FTOE, Fractional tissue oxygen extraction; CrSO2, Cerebral tissue oxygen saturation; S100A12, S 100 calcium binding protein a 12; I-FABP, Intestinal fatty acid binding proteins; IAP activity, Intestinal alkaline phosphatase activity; SAA, Serum amyloid A; CBG, Cytosolic β- glucosidase; DEGs, Differentially expressed genes; IAIP, Inter-alpha inhibitor proteins; AMC, Absolute monocyte counts.

Abdominal X-ray is the most common diagnostic tool for NEC, but the disease is already in the progression stage by the time when any manifestation can be detected in the X-ray. This, however, seems late. Since the onset of NEC is occult and early clinical manifestations are not specific, early detection of NEC is challenging for clinicians. Since immature intestinal barrier function, hypoxia-ischemia, and flora imbalance are involved in the occurrence and development of NEC (4, 5), it might be feasible to detect NEC from those aspects, by utilizing ultrasound, near-infrared spectroscopy (NIRS), biomarker monitoring, and intestinal microbiota diversity. This article reviews the tools and markers that are potential for predicting or diagnosing NEC at an earlier stage (Figure 1).

## Ultrasound

In recent years, ultrasound has been widely used in the diagnosis and monitoring of NEC (6). It can detect NEC-related symptoms at the early stage (7), with advantages such as portability, non-invasiveness, and real-time result. NEC patients show portal venous gas, pneumatosis intestinalis, intestinal hypoperfusion, focal fluid collection, and free intraperitoneal gas during ultrasound examination (8–10).

Studies have shown that abdominal ultrasound may predict the occurrence of NEC by monitoring the blood flow of the superior mesenteric artery (SMA). In a prospective study, Guang et al. placed an ultrasonic probe under the xiphoid to monitor the SMA in 104 newborns within 12 h after birth. The results showed that the differential velocity [peak systolic velocity (PSV) minus end-diastolic velocity (EDV)] of the SMA had a high sensitivity (0.875) and specificity (0.604) for predicting NEC with a cut-off value of 34.835 cm/s (11). This study suggests that the increase in differential velocity may be a predictor of NEC. Murdoch et al. found that the risk of NEC was positively correlated with the SMA resistance index (PSV—EDV)/(PSV) on the first day after birth, while the risk of NEC decreased in neonates with an increased SMA end-diastolic blood flow velocity (12). SMA blood flow in a rabbit model of NEC displayed the same changes (13). In a case-control study, 62 newborns were examined by Doppler ultrasound; the results showed that an SMA resistance index >0.75 and a pulsatility index [(PSV—EDV)/mean velocity] >1.85 could distinguish the NEC and control groups (14). Abnormal blood flow in the SMA may induce intestinal ischemic injury, which can then lead to the occurrence of NEC.

Contrast-enhanced ultrasound (CEUS) can be used to assess intestinal perfusion and motor degradation. CEUS enhances blood flow and tissue echo through contrast microbubbles. When CEUS is performed to evaluate intestinal wall and SMA perfusion, contrast agents are usually injected intravenously, but there is no dose guideline at present (15). Studies have shown that CEUS may be applied to many gastrointestinal diseases (16). Since high-frequency oscillator impedes the detection of intestinal perfusion by Doppler ultrasound, a case report used CEUS to evaluate intestinal perfusion in children undergoing high-frequency oscillator ventilation, which confirmed the absence of intestinal perfusion. Subsequently, laparotomy exploration revealed total intestinal necrosis, which indicated that CEUS could be used when Doppler ultrasound failed to evaluate blood perfusion (17).

## NIRS

NIRS is a non-invasive tool to monitor tissue blood oxygen (18). Animal studies have found that NIRS can detect instant and persistent gastrointestinal hypoperfusion (19) and that monitoring tissue oxygen saturation (rSO2) in infants by NIRS is feasible (20). Therefore, NIRS may be able to predict NEC through the continuous monitoring of intestinal and cerebral oxygenation.

Studies have demonstrated impaired visceral oxygenation before the onset of symptoms of NEC (21, 22), by placing the sensors below the umbilicus or over the right lower abdomen. The average splanchnic oxygen saturation of patients with NEC during the first postnatal week was significantly lower than that of non-NEC controls (23). Abdominal oxygen saturation ≤ 56% has been suggested to be predictive of NEC, with a sensitivity of 86% and specificity of 64% (24).

Fractional tissue oxygen extraction (FTOE) reflects the relationship between tissue oxygen supply and oxygen consumption. FTOE is calculated based on the data measured by NIRS: FTOE = (oxygen saturation—rSO2)/ oxygen saturation. A study found that the intestinal FTOE of children with NEC would increase 2 days before the onset of symptoms, suggesting that FTOE may also be predictive of NEC (22). Increased FTOE may be a compensatory response to hypoxia to maintain aerobic metabolism (22, 25). Interestingly, Schat et al. predicted abdominal diseases by monitoring brain oxygenation and found that the prevalence of NEC in infants with cerebral rSO2 <70% within 48 h after birth was significantly higher than that in infants with cerebral rSO2 ≥70% [odds ratio (OR): 9.00] (22). Cerebral rSO2 is a marker of systemic perfusion, and hypoxia-ischemia is closely related to the occurrence of NEC (22, 26).

Currently, NIRS is mainly used to monitor brain oxygen saturation in children, so NIRS algorithms are mostly based on the brain and are not fully applicable to the abdomen (27). Since there are differences in oxygenation in different parts of the viscera, it is difficult to obtain accurate data (27, 28). Abdominal oxygen saturation has more variation than that of the brain and kidney (29), which also limits the clinical application of NIRS.

## Biomarkers

Many studies have proposed diagnostic and predictive biomarkers for NEC (Tables 1–3). Biomarkers are typically obtained from serum, fecal, or urine samples. Therefore, they are not affected by the preference or skills of the performers. Besides, the tests will be very easy and non-invasive when fecal or urine samples are used. However, most of them are not as specific as ultrasound presentations. Different methods or essays may result in diverse reference values that are impossible to integrate into a universal reference. Therefore, more studies are needed to investigate the markers for the prediction and early diagnosis of NEC.

**Table 1:** Fecal biomarkers of NEC.

**Biomarker**	**References**	**Subject (number)**	**Sample**	**Assay**	**Predictive/diagnostic value**
Calprotectin	(30)	Humans (206)	Feces	ELISA	P
	(31)	Humans (132)	Feces	ELISA	P
	(32)	Humans (120)	Feces	ELISA	D
	(33)	Humans (568)	Feces		D
	(34)	Humans (110)	Feces	ELISA	D
S100A12	(35)	Humans (145)	Feces	ELISA	P, D
IAP	(36)	Rats	Tissue	Colorimetric assay	
	(37)	Humans (136)	Feces	Fluorometric detection	D

ELISA, Enzyme-linked immunosorbent assay; P, Predictive value; D, Diagnostic value; S100A12, S 100 calcium binding protein a 12; IAP, Intestinal alkaline phosphatase.

**Table 2:** Urine biomarkers of NEC.

**Biomarker**	**References**	**Subject (number)**	**Sample**	**Assay**	**Predictive/diagnostic value**
I-FABP	(38)	Humans (37)	Urine	ELISA	D
	(39)	Humans (78)	Urine, Blood	ELISA	D
	(40)	Humans (37)	Urine, Blood	ELISA	D
	(41)	Humans (140)	Urine	ELISA	P
	(42)	Humans (62)	Urine	ELISA	P
	(43)	Humans (35)	Urine	ELISA	
SAA	(38)	Humans (37)	Urine	ELISA	D
	(44)	Humans (62)	Urine	ELISA	D
	(45)	Humans (29)	Urine	ELISA	D

I-FABP, Intestinal fatty acid binding proteins; ELISA, Enzyme-linked immunosorbent assay; D, Diagnostic value; P, Predictive value; SAA, Serum amyloid A.

**Table 3:** Blood biomarkers of NEC.

**Biomarker**	**References**	**Subject (number)**	**Sample**	**Assay**	**Predictive/diagnostic value**
I-FABP	(39)	Humans (78)	Urine, Blood	ELISA	D
	(40)	Humans (37)	Urine, Blood	ELISA	D
IAIP	(46)	Humans (51)	Blood	ELISA	D
	(47)	Humans (53)	Blood	ELISA	D
DEGs	(48)	Piglets (129)	Blood	qPCR	D
	(49)	Rats	Tissue	RNA-Seq	D
	(50)	/	/		D
	(51)	Humans (301)	Blood	Microarray, qPCR	D
SAA	(52)	Humans (144)	Blood	Immunonephelometric method	D
	(53)	Humans (154)	Blood	Immunoassay	D
CBG	(54)	Rats	Blood	ELISA	P, D
	(55)	Humans (82)	Blood	ELISA	D
	(56)	Humans (205)	Blood		D
Surrogate markers of NETosis	(57)	Mice, humans	Blood, tissue	ELISA	D
	(58)	Mice, humans (76,9)	Blood	Immunohistochemical staining	D
AMC	(59)	Humans (326)	Blood		D
	(60)	Humans (105)	Blood		D

I-FABP, Intestinal fatty acid binding proteins; ELISA, Enzyme-linked immunosorbent assay; D, Diagnostic value; IAIP, Inter-alpha inhibitor proteins; DEGs, Differentially expressed genes; qPCR, Quantitative polymerase chain reaction; RNA-Seq, RNA sequencing; SAA, Serum amyloid A; CBG, Cytosolic β- glucosidase; P, Predictive value; AMC, Absolute monocyte counts.

### Calprotectin

Calprotectin belongs to the S100 family and is mainly produced by neutrophils (61). It is involved in the innate immune response by activating Toll-like receptors (62), which participate in the pathogenesis of NEC. Therefore, some scholars believe that calprotectin is a biomarker for the early prediction of NEC (30, 31).

Elevated fecal calprotectin in the early life of newborns may be related to NEC (32). For preterm infants with a gestational age ≤ 26 + 1 weeks, the sensitivity and specificity of fecal calprotectin at 24 h before the onset of clinical symptoms were 0.89 and 0.87, respectively (30). The fecal calprotectin concentration of patients with NEC was significantly increased by 2.1 times (63). A meta-analysis of ten studies showed that the sensitivity, specificity, diagnostic odds ratio (DOR), and area under the curve (AUC) for the early diagnosis by fecal calprotectin were 0.86, 0.79, 34.78, and 0.92, respectively (33). In addition, Thibault et al. reported that the combined regimen of calprotectin and lipocalin-2 was more favorable for the prediction of NEC within the first 10 days before the onset of NEC than either marker independently (31).

There is some controversy on this. Some studies suggest that fecal calprotectin has a large variation between individuals (64). A prospective case-control study reported that there was no statistical difference in calprotectin concentration between the NEC group and the control group at the 6–8 days (*P* = 0.80), the 3–5 days (*P* = 0.80), and within 48 h (*P* = 0.80) before NEC was suspected (64). Additionally, Goold et al. analyzed two cut-off values of calprotectin for diagnosing NEC (226 and 247 μg/g), both with low diagnostic efficiency (34). According to the research above, fecal calprotectin remains to be further explored to guide the early prediction and diagnosis of NEC.

### S100A12

Human S100A12 up-regulates the expression of inflammatory genes by interacting with Toll-like receptor 4 (65). It is also involved in ischemia-reperfusion injury through the activation of ERK signaling (66), which is considered to be involved in the pathogenesis of transfusion-related NEC. A study found that the level of S100A12 in the feces of infants with suspected NEC was higher than that of infants without gastrointestinal diseases; the sensitivity, specificity, and positive and negative predictive values of fecal S100A12 for NEC detection were 70%, 68%, 37%, and 89%, respectively (35).

### Intestinal alkaline phosphatase

IAP is expressed in the gastrointestinal tract and plays a role in maintaining the homeostasis of the intestinal environment (67). Animal experiments have shown that the IAP activity of NEC pups was 0.18 U/mg, which was significantly lower than that of healthy controls (0.57 U/mg). Furthermore, IAP activity increases after the removal of NEC stressors (36). Heath et al. observed similar results in human NEC patients and believed that IAP could not only diagnose NEC but also predict the severity of the disease (37). In this study, the fecal protein IAP activity of children with severe NEC (characterized by radiologic evidence of pneumatosis intestinalis and/or portal venous gas) and suspected NEC (abnormal clinical and laboratory findings without evidence of pneumatosis intestinalis or portal venous gas) was 183(56-507) μmol/min/g and 355 (172–608) μmol/min/g, respectively, and the accuracy of assessing severe NEC by IAP activity was 0.76 (95% CI: 0.64–0.86; *P* < 0.001) (37).

### Intestinal fatty acid binding proteins

Fatty acid binding proteins (FABPs) have the pleiotropic function to maintain healthy tissue homeostasis and participate in disease pathogenesis (68). I-FABP is a 15-kDa cytoplasmic protein expressed mainly by intestinal cells located at the top of the intestinal villi. This protein is released when the intestinal tissue is ischemic or damaged. The I-FABP response to intestinal hypoperfusion is related to the pathogenesis of NEC (69). Studies suggest that I-FABP levels in the urine and blood can be used in the prediction and early diagnosis of NEC (38–41).

Increased urinary I-FABP in infants is associated with subsequent NEC (38). A cohort study showed that I-FABP >13.3 ng/mL could predict NEC with 60% sensitivity and 78% specificity seven days before the onset of symptoms, and I-FABP >13.9 ng/mL was found to have a 65% sensitivity and 84% specificity within 3 days before NEC onset (41). Urinary I-FABP/creatinine >10.2 pg/nmol had a sensitivity of 100% and specificity of 95.6% 1 day before NEC onset (42).

The urinary and plasma level of I-FABP in patients with NEC was reported to be significantly higher than that in other infants (40, 43). Schurink et al. determined that the cut-off values for NEC diagnosis within 8 h after onset were 9 ng/mL (plasma I-FABP) and 218 ng/mL (urinary I-FABP), and the corresponding likelihood ratios were 5.6 and 5.1, respectively (40). FABP can also help to predict the need for surgical treatment (39, 70, 71). I-FABP distinguishes patients who need conservative treatment from those that need surgical treatment in the early stage of NEC with the cut-off values of 19 ng/mL (plasma I-FABP) and 232 ng/mL (urinary I-FABP) (40).

I-FABP is also a potential marker for distinguishing NEC from sepsis. In a study of 42 infants, the I-FABP concentration in patients with NEC is significantly higher than that in patients with sepsis (72). Another study combines plasma I-FABP, liver-type FABP (L-FABP), and intestinal trefoil factor as LIT score (0–9) to differentiate NEC from sepsis. The LIT score of children with NEC is significantly higher than that of children with septicemia (*P* = 0.001) (73).

Although there have been many studies on I-FABP as an early diagnostic and predictive marker of NEC, the cut-off value for the diagnosis and staging of NEC has not been determined due to the inconsistency of the studies. Therefore, multicenter studies with larger sample sizes are needed to aid clinicians in decision making.

### Inter-alpha inhibitor proteins

IAIP has extensive anti-inflammatory activity and participates in neutralizing extracellular histone cytotoxicity (74). A prospective observational study of 34 newborns shows that the plasma IAIP level in patients with NEC decreases (*P* < 0.0001) (46). Shah et al. suggests that IAIP <207 mg/L has a high diagnostic value for NEC (AUC: 0.98, sensitivity: 100%, specificity: 88.2%). On this basis, IAIP can help differentiate NEC from spontaneous intestinal perforation (47). However, IAIP is not specific for predicting NEC as it also decreases in the plasma of patients with sepsis (75). Stober et al. have shown that IAIP can relieve endothelial inflammation in sepsis and protect endothelial cells from damage caused by activation of C5a (76), but there is no research on its effect against NEC.

### Differentially expressed genes

Changes in gene expression of the whole blood are mainly related to severe NEC, and their expression in mild NEC changes slightly (48). Compared to normal controls, 53 circular RNAs have been found to change in the NEC group (49). In recent years, studies have identified some DEGs in infants with NEC. The expression of micro *RNA (miR)-223* and *miR-451a* is up-regulated in patients with NEC at disease onset (50, 63) and *miR-223*/nuclear factor I-A axis may play an important role in the pathophysiology of NEC by aggravating inflammation and tissue damage (77). A meta-analysis shows that *miR-429/200a/b* and *miR-141/200c* clusters are poorly expressed in patients with NEC. They can down-regulate target genes related to NEC, and be used as potential biomarkers for early detection (50). Furthermore, a prospective cohort study found that *miR1290* might be used as a specific marker for the early diagnosis of NEC (sensitivity: 0.83, specificity: 0.96, DOR: 127.50). It has high diagnostic efficiency and can effectively distinguish NEC from sepsis (51).

### Serum amyloid A

SAA is mainly derived from hepatocytes. Its expression increases rapidly in case of inflammatory response. Serum SAA level increases significantly after infection, trauma, and other stimulation (78).

In a study including 144 infants, the SAA level in blood samples of NEC patients is significantly higher than that of healthy controls (43.2 ± 47.5 mg/dl vs. 3.2 ± 3.4 mg/dl, *P* < 0.05) at the time of NEC diagnosis (52). SAA is more common in predicting NEC together with other markers. A prospective cohort study differentiates NEC from sepsis using apoSAA score, which is calculated from plasma apolipoprotein C2 (apoC2) and SAA; the results show that the apoSAA score is helpful in the early diagnosis of NEC and the differentiation of NEC from sepsis (53). A combination of liver FABP, I-FABP, and SAA may indicate portal venous gas in NEC (38). However, Reisinger et al. find that the combination of urinary SAA and I-FABP can not improve the diagnostic accuracy of NEC compared with I-FABP alone (44). Urinary SAA in the NEC surgery group is significantly higher than that in the conservative group before surgery (38). The cut-off value was 34.4 ng/mL (sensitivity: 83%; specificity: 83%) (45). Therefore, SAA combined with other markers is expected to promote the early diagnosis of NEC.

### Cytosolic β-glucosidase

CBG is a member of the cellulase family, which is widely distributed in nature and has also been detected in the human body. CBG is a hydrolytic enzyme distributed in intestinal epithelial cells. Animal experiments find that serum CBG is significantly elevated in ischemic injury caused by arterial occlusion or closed intestinal obstruction (79). Dimmitt et al. measured the serum CBG activity in an NEC rat model and found that serum CBG activity increased before the onset of transmural injury, suggesting that CBG was meaningful for early monitoring of intestinal ischemic injury (54). Subsequently, an increase in serum CBG in neonates with NEC is also confirmed (55). Serum CBG activity of NEC neonates is higher than that of non-NEC neonates, and the cut-off value of 15.6 mU/mg may distinguish neonates with NEC from healthy neonates at the early stage (56). Therefore, CBG can provide potential evidence for the early diagnosis of NEC.

### Surrogate markers of NETosis

Neutrophils participate in the NEC inflammatory response by phagocytosis, degranulation, or neutrophil extracellular traps (NETs) (80). NETs occur in both human and mouse NEC tissue (57). The formation of NETs is accompanied by the death of neutrophils, known as NETosis (81). In recent years, studies have shown that NETs play a role in the intestinal inflammatory response containing NEC (58, 82–84). Vincent et al. report that serum substitute markers, such as cfDNA and DNase of NETosis may predict the occurrence of NEC in mouse models; cfDNA and DNase increase significantly with the induction time of the NEC model, and receiver operating curve analysis shows that the cut-off value of cfDNA is 1,250 ng/mL, which has a sensitivity and specificity of 100% for NEC (58). Serum substitutes of NETosis are expected to become biomarkers for the early diagnosis of NEC; however, the evidence is limited. More studies establishing a relationship between NEC and NETosis are still needed.

### Absolute monocyte counts

Compared with the baseline value, the AMC of children with NEC significantly decreased. In infants with feeding intolerance, AMC decreases by more than 20%, and the sensitivity and specificity for predicting NEC are 0.70 and 0.71, respectively (59). AMC also differs at different stages of NEC. The AMC of patients with NEC stage III decreases by 81.9%, which is significantly lower than that of II-NEC (44.5%) (60). Thus, AMC may be a potential biomarker for the early diagnosis and severity assessment of NEC.

## Microbiota

The normal intestinal flora resists pathogens and participates in the intestinal immune response (85). Intestinal microflora disorders are present before and after NEC (86–88), indicating that microbial communities are related to the occurrence and development of NEC.

A study analyzes the fecal samples of children with NEC using 16 s rRNA and metagenomic sequencing technology and finds that the intestinal microbial diversity of children with NEC decreases (87, 89). Hosfield et al. also demonstrate the association between intestinal microbial diversity and NEC in an animal model (86). In contrast, no difference in intestinal microbial diversity is found in a case-control study of 32 newborns, possibly due to factors such as a small sample size (90). Children with NEC not only have changes in microbial diversity, but they also have significant changes in the composition of the flora. The intestinal flora of healthy infants consists mainly of *Bifidobacterium* (91). The relative abundance of *Bifidobacterium* and *Lactobacillus* in patients with NEC decreases (86, 87, 90, 92), whereas the abundance of Escherichia and Klebsiella increases (86). Similar changes also occur before the onset of NEC (88, 89, 92). Therefore, monitoring neonatal intestinal microbiota may be a potential method for the prediction of NEC. However, its application is limited due to the significant differences in intestinal flora composition among different regions (93).

Maternal milk affects the establishment of the early neonatal intestinal microbiota (94). Human milk oligosaccharides are important components that participate in the regulation of intestinal microbiota by inhibiting pathogen adhesion, growth, and reproduction in the intestinal tract (95). Masi et al. analyzed 19 oligosaccharides of mother's breast milk and found that the concentration of disialyllacto-N-tetraose in oligosaccharides of mother's breast milk of children with NEC decreased (*P* < 0.001). Univariate analysis shows that the concentration of disialyllacto-N-tetraose is 241 nmol/mL, which can distinguish NEC from non-NEC infants with an accuracy of 91% (92). Another case-control study also supports this conclusion and indicates that oligosaccharides are protective factors against NEC.

Volatile organic compounds (VOCs) in the feces reflect the composition of intestinal microflora and the interaction between microbiota and the host. Fecal VOCs assessed by electronic nose can be used to predict microbial composition (96). Fecal VOC profiles change significantly in inflammatory bowel disease (97) and colitis (98). In NEC experimental animals, fecal VOC profiles are different from those of the control group (86). Moreover, a multicentre prospective study reports that children with NEC can be distinguished from the control group by fecal VOC profiles at 2–3 days (AUC: 0.77 ± 0.21; sensitivity: 83%; specificity: 75%) and 0–1 days (AUC: 0.99 ± 0.04; sensitivity: 89%; specificity: 89%) before the onset of clinical symptoms of NEC (99). Wright et al. also suggest that VOCs can be detected early before NEC occurs (100). Fecal VOC may be a promising marker for the prediction of NEC.

## Limitation

We only reviewed the prediction and early diagnosis of NEC with a single tool, and did not discuss the combination of tools. In addition, we have not conducted much analysis on the applicability of each tool.

## Summary and conclusion

Although there have been numerous attempts in the literature to develop new techniques or summarize existing regimens to predict the incidence of NEC or diagnose NEC at a very early stage, there have been no established regimen that can be universally accepted. Ultrasound and NIRS mainly detect changes in blood flow and oxygenation, which are helpful in the early prediction of NEC. Novel biomarkers, including calprotectin and I-FABP show great potential to become an independent or part of a complex regimen. However, some other biomarkers are still facing a long way from experimental studies to clinical practice. The intestinal microbiota has been profoundly investigated, but our current achievements still cannot guarantee its wide application. Since the prediction and timely diagnosis of early-stage NEC may significantly benefit NEC victims, future investigations and co-operations are still invaluable.

### Method

Data for this review were identified by searches of PubMed, we first searched through “diagnosis” and “necrotizing enterocolitis” and screened out the literature related to the early diagnosis of NEC through abstracts and rough reading of the literature. Then we searched through “prediction” and “necrotizing enterocolitis ” and screened out the literature predicting NEC. Finally, we searched through “ultrasound,” “near infrared spectroscopy,” “biomarkers,” “microbiota,” “diagnosis,” “prediction” and “necrotizing enterocolitis” and other related words were searched to avoid missing relevant literature.

## Author contributions

YS and TL designed the overall study. SW drafted the manuscript of the article. SW and SD performed the systematic literature search. YS revised the structure and logic of the article. All authors reviewed the manuscript critically and approved the final version of manuscript.

## Funding

This work was supported by National Natural Science Foundation of China (82171709 and 81801500), the 345 Talent Project of Shengjing Hospital (M0275 and M0279), and Key R&D Guidance Plan Projects in Liaoning Province (2020JH1/10300001).

## Conflict of interest

The authors declare that the research was conducted in the absence of any commercial or financial relationships that could be construed as a potential conflict of interest.

## Publisher's note

All claims expressed in this article are solely those of the authors and do not necessarily represent those of their affiliated organizations, or those of the publisher, the editors and the reviewers. Any product that may be evaluated in this article, or claim that may be made by its manufacturer, is not guaranteed or endorsed by the publisher.
